# Efficacy of Intrauterine Injection of Granulocyte Colony Stimulating Factor (G-CSF) on Treatment of Unexplained Recurrent Miscarriage: A Pilot RCT Study

**Published:** 2017

**Authors:** Simin Zafardoust, Mohammad Mehdi Akhondi, Mohammad Reza Sadeghi, Afsaneh Mohammadzadeh, Atousa Karimi, Sheyda Jouhari, Soheila Ansaripour

**Affiliations:** Reproductive Biotechnology Research Center, Avicenna Research Institute, ACECR, Tehran, Iran

**Keywords:** Granulocyte colony-stimulating factor, Intrauterine injection, Recurrent miscarriage

## Abstract

**Background:**

Endometrium undergoes several changes in structure and cellular composition during pregnancy. Granulocyte Colony-stimulating Factor (GCS-F) is an important cytokine with critical role in embryo implantation and pregnancy. The aim of the present study was to evaluate the impact of intrauterine injection of G-CSF in patients who suffer from unexplained recurrent miscarriage (RM).

**Methods:**

In the present randomized clinical trial, a total of 68 patients were randomly allocated into two study groups including intrauterine G-CSF (n=23, 300 *μg*) injection and control group (n=27, no G-CSF injection). Eighteen out of 68 patients were excluded from the final analysis due to different reasons. All patients were in Ovulation Induction (I/O) cycle. In G-CSF group, intrauterine injection of G-CSF was done twice in the cycle. All enrolled patients were under 40 years old and had at least two unexplained pregnancy losses. Pregnancy was evaluated by titer of βhCG, presence of gestational sac (implantation) and fetal heart rate (clinical pregnancy) was assessed by vaginal ultrasonography. Student’s T test and Mann-Whitney U were used for analysis. The p≤0.05 was determined as statistically significant.

**Results:**

No significant differences were observed between the two study groups when the rates of chemical pregnancy (26.1% *vs*. 29.6%, p=0.781), implantation (26.1% *vs*. 22.2%, p=0.750), clinical pregnancy (17.4% *vs*. 11.1%, p=0.689) and abortion (33% *vs*. 37.5%, p=0.296) were compared.

**Conclusion:**

In our study, no significant difference was observed between the two study groups when the rates of chemical pregnancy, implantation, clinical pregnancy and abortion were compared.

## Introduction

Recurrent Miscarriage (RM) is a quite common disorder in about 15% of all abortions following clinically recognized pregnancies (1). Recurrent Miscarriage (RM), defined as 3 consecutive pregnancy losses prior to 20 weeks from the last menstrual period, affects approximately 1% to 2% of women (2). Etiology of RM has not been recognized completely. Some cases are related to genetic, endocrine, anatomical and infectional factors (3–5). It is assumed that about 20% of cases are related to autoimmune disorders and about 40–50% of cases are unexplained RM G-CSF (Granulocyte Colony-stimulating Factor) is a glycoprotein that is secreted from endothelial cells, macrophages and some other immune system cells (6). This growth factor is encoded by a gene on human chromosome 17 and acts as a ligand for its specific receptors that are encoded by the same chromosome (7, 8). Effects of G-CSF in several disease such as Alzheimer’s disease (9), acute graft-versus-host disease (10), Crohn’s disease (11, 12) and heart disease (13, 14) have been investigated. In recent years, it is introduced as a treatment option for women with unknown RM (15). Effects of GCSF injection in patients suffering from recurrent pregnancy loss have been studied in some studies and it seems that transvaginal endometrial perfusion with G-CSF might be helpful for improvement of implantation rate among patients with thin endometrium and patients under IVF/ET or ICSI/ET (16). Levels of granulocyte-macrophage colony stimulating factor (GM-CSF) and vascular epithelial growth factor (VEGF) in cervical mucus (CM) have been shown in relationship with endometrial receptivity in the IVF cycle (17).

The important factor regarding recurrent abortion is recognizing the possible treatments that are beneficial. Other studies have not used intrauterine G-CSF in patients suffering from unexplained RM. The aim of the present study was to evaluate the impact of intrauterine injection of G-CSF in patients suffering from unexplained RM for decreasing the abortion rate.

## Methods

### Study population:

The present randomized clinical trial was performed at Avicenna Infertility Clinic affiliated to Avicenna Research Institute, Tehran, Iran, during a 12-month period from January 2013 to January 2014.

This study was a randomized clinical trial (Registration ID in IRCT: IRCT2013012211430N3) with a total of 68 patients who were under 40 years old and had experienced at least one of the following criteria including at least two times of sequential unexplained abortions or three times of non-sequential unexplained abortions. They had normal values for FBS, TSH, normal karyotyping of couple and FSH under 10. All patients were checked for absence of uterine anomalies, infectious diseases and dysfunctions in endocrine system. Negative results for lupus anticoagulant, anticardiolipin antibodies (IgG, IgM), β2 glycoprotein antibodies (IgG, IgM) and infectious tests (Toxoplasma IgM, Cytomegalovirus IgM, VDRL, HCV and Rubella IgM) were inclusion criteria. All included subjects were normal regarding lab test of Factor V Leiden, Factor II (prothrombin), PAI-I and Proteins C and S (two vitamin K-dependent plasma proteins).

Exclusion criteria in the present study were current cancer and presence of paternal or maternal chromosomal aberration. Patients with uncontrolled diabetes and thyroid disorders, FSH≥10 and TPO≥500 were also excluded due to possible effects of these factors on their abortion.

### Random allocation:

In this study, computer-generated randomization list was used for randomization. A complete history evaluation and physical examination were conducted for all the participants by the attending gynecologist. Included subjects were randomly allocated into two study groups as previously mentioned.

### Intervention:

Aspirin (80 *mg/day-oral*, Parsdarou, Iran) was administered for both groups from initiation of I/O (Ovarian Induction) and heparin (5000 *U/each* 12 *hr*-S.C., Caspian, Iran) when the gestational sac was observed by vaginal ultrasound. Ovulation induction was performed using clomiphene citrate (50 *mg*/2 pills a day: Iran Hormone, Iran) from day 3 to day 7 of menstrual cycle to stimulate development of one or more mature follicles. All patients were subjected to intra-muscular (IM) injection of a vial of human menopausal gonadotropin (hMG, Menogan: Ferring, Switzerland) on the 8th day of menstrual cycle. After 4–5 days, vaginal ultrasound was conducted and if an appropriate follicle (≥18 *mm*) was observed, the intervention started. Intervention group subjects received 300 *μg* of G-CSF (Filgrastim, Switzerland), transvaginally by using an insemination catheter (Prodimed, France). After lying in absolute rest for 30 *min*, patients were discharged. For control group, no G-CSF injection was admitted. In G-CSF group, intrauterine injection of G-CSF was performed twice in a cycle. After the first injection of G-CSF, muscular injection of HCG (10000 *IU*, Choriomon, IBSA, Switzerland) was administered and couples had intercourse every other day until one week. If the follicle’s size was not suitable, a second muscular injection of hMG was tabled. The second intrauterine injection of G-CSF was conducted 7 days after HCG injection (because it is the estimated day for implantation).

Three criteria were considered for evaluation of pregnancy. The first one was titer of βhCG (16 days after hCG injection). The second criterion was the presence of gestational sac (implantation) that was assessed by vaginal ultrasonography (Honda 2000) at 6–7 weeks of pregnancy and finally the last but not least criterion was clinical pregnancy that was defined as the confirmed detection of fetal heart rate (FHR).

### Control group:

For control group, no placebo was used and all the stages of procedure were the same as treatment group but the intrauterine injection was eliminated from the process.

### Follow-up:

Among pregnant subjects, follow-up has been done until the 20th week.

### Ethics:

This project was approved by Research Ethics Council of Avicenna Research Institute (Code: 91-021) and IRCT (IRCT2013012211430 N3). A standard written informed consent was obtained from each patient before participating in the study.

### Statistical analysis:

Eighteen out of 68 patients were excluded from the final analysis due to different reasons. From intervention group, seven patients didn’t refer for the second round of injection. Two patients were excluded due to the ineligible response to I/O treatment and finally 2 patients were excluded because of insufficient contact information and impossibility of follow-up. Among control group, seven subjects were excluded. Three patients because of insufficient response to I/O treatment were excluded, three subjects didn’t refer after beginning of the cycle and lastly in one case, the follow-up was not completed.

Data analysis was performed using SPSS software version 11.5. Quantitative variables such as age, endometrium thickness, number of follicles and FSH levels were compared between groups by Student’s T test or Mann-Whitney U in case of ordinal variables or non-normal distribution. Normal distribution of quantitative variables was tested using One-Sample Kolmogorov-Smirnov Test. Descriptive data were presented as Mean±SD. Qualitative variables such as existence of gestational sac, endometrium pattern and detection of fetal heart rate were assessed by chi-square test and in some cases, Fisher’s exact test was used. The p-value under 0.05 was determined as statistically significant.

## Results

Eighteen out of 68 patients were excluded from the final analysis due to different reasons. From intervention group, seven patients didn’t refer for the second round of injection. Two patients were excluded due to the ineligible response to I/O treatment and finally 2 patients were excluded because of insufficient contact information and impossibility of follow-up. Among control group, seven subjects were excluded. Three patients because of insufficient response to I/O treatment were excluded, three subjects didn’t refer after the beginning of the cycle and lastly in one case, the follow-up was not completed.

From a total of 50 patients that have completed the clinical trial procedure, 23 persons (46%) were in intervention group and 27 individuals (54%) were in control group.

Background characteristics of intervention and control group were compared. Mean age in control group was 30.2±4 versus 31.6±5.6 for intervention group. No significant difference was found in age (P: 0.082) status. Comparison of the number of previous abortions among two study groups, FSH on 2nd or 3rd day of menstrual bleeding and BMI also revealed no statistically significant difference.

Endometrial thickness and the number of follicles>16 *mm* in the last ultrasonography in the cycle of I/O, were observed among 21 out of 23 patients in intervention group and 26 out of 27 patients in control group. The differences between the two study groups were not statistically significant (Table 1).

**Table 1.** Demographic characteristics of patients in G-CSF and control groups

**Parameter**	**G-CSF (n=23)**	**Control (n=27)**	**p-value**
**Age**	30.2±4	31.6±5	0.082
**BMI**	23.4±2	25.6±3	0.180
**Number of previous abortions**	3.0±1	2.7±0.8	0.845
**FSH**	7.2±2	7.0±2	0.150
**Endometrial thickness**	5.7±1	6.5±1	0.126
**Number of follicles**	1.3±0.5	1.7±0.7	0.068

The level of βhCG was tested among participants of the two groups. Among 23 subjects of intervention group, 6 (26.1%) positive tests were reported and in control group, 8 out of 27 subjects (29.6%) had positive βhCG test. The difference between the two groups was not statistically significant (P: 0.781).

Development of gestational sac, occurrence of clinical pregnancy and abortion were compared. Between subjects who had positive results for βhCG test, gestational sac was developed among all pregnant individuals in the first group (Intervention) but 2 out of 8 pregnant patients of the control group (25%) didn’t develop the gestational sac. This difference was also non-significant (p=0.491). Then, in the first group (Intervention), 6 patients (26.1%) and in control group, 6 patients (22.2%) had gestational sac (p=0.750).

Clinical pregnancy was also compared between the two groups. There was no significant difference between two study groups regarding clinical pregnancy (17.4% *vs*. 11.1%, Fisher’s exact test, p=0.689). Rates of abortion among two groups were compared and no significant difference in this issue was found (33% *vs*. 37.5%, Fisher’s exact test, p=0.296) (Table 2).

**Table 2.** Outcome measures of patients in G-CSF and control groups

**Parameter**	**G-CSF**	**Control**	**p-value**
**βhCG (%)**	26.1	29.6	0.781
**Gestational sac (%)**	26.1	22.2	0.750
**FHR (%)**	17.4	11.1	0.689 ^*^
**Abortion (%)**	33	37.5	0.296 ^*^

* Fisher’s Exact Test

## Discussion

In our study, no significant differences were observed between the two study groups when the rates of chemical pregnancy, implantation, clinical pregnancy and abortion were compared.

It is estimated that T helper 1 (Th1) and T helper 2 (Th2) cells play contradictory roles in embryo development. Th2 cells are helpful for development of new embryos alternatively and it is assumed that Th1 cells promote some processes which are harmful for embryo (19). All things considered, treatments targeting deficient immune system in RM patients may help down regulate its adverse effects on the embryo implantation and persistence of embryo development (20).

Killer-cell immunoglobulin-like receptors (KIRs) are expressed at the site of placentation by Natural Killer cells (NK cells). Specific ligand of these receptors is called human leukocyte antigen-C (HLA-C). Association of polymorphisms of these two genes with susceptibility to pre-eclampsia has been approved. In addition, pre-eclampsia and RM have shared the defective placentation in their pathogenesis, so in this regard, a reliable steadiness in inhibition and activation of NK cells is needed to achieve an effective placentation (21).

Considering the effects of immunological factors on risk of recurrent miscarriage (21, 22), it can be said that modulation of this system may affect the process of abortion and consequently reduce the rate of unexplained miscarriage. So when the purpose is to study the etiology of recurrent miscarriage, not only should chromosomal and uterine abnormalities or autoimmune disorders be considered for evaluation but also inflammatory processes stimulated by cytokines released immune system cells should be studied (19, 21). Administration of immunomodulatory factors such as G-CSF and TNF-α blockers for treatment of recurrent pregnancy loss has been the focus of many studies recently. There is some evidence regarding effectiveness of inhibitors of TNF-α cytokine (23) and much more evidence for G-CSF (15, 24).

The results of the present study showed no significant increase in the pregnancy rate of RM patients treated by intrauterine injection of G-CSF in comparison with control group. Furthermore, the true mechanism with which G-CSF is useful for patients suffering from RM should be under scrutiny.

There are some studies that assume serum and follicular fluid G-CSF levels could be considered as suitable predictors for IVF outcomes. The main explanations for this claim are the role of G-CSF in follicle development and ovulation and also its effects on the process of implantation (25). There are also some proofs for usage of follicular fluid (FF) G-CSF among individual embryos for selection of better ones for implantation (26).

G-CSF therapy has been applied to surmount several gynecological disorders such as RM, Repeated Implantation Failure (RIF) and follicle development deficiencies so far. Scarpellini et al. selected a group of women in which conventional therapies for RM were failed and conducted a RCT for testing G-CSF treatment by subcutaneous administration of 1 *μg/kg/day*. According to Scarpellini et al., administration of 100000 *IU/kg/day* until 9th week of pregnancy could have a positive effect on the live birth rate and the βHCG rate in women suffering from unexplained RM (24). These results are consistent with the findings of our study so the mechanism behind this process is remained unclear. The authors concluded that although there is not enough data to confirm the role of G-CSF in increasing the chance of childbirth among patients suffering from RM, there is some evidence in relation to non-toxicity of this immune modulator substance in pregnant women and significant increasing in levels of β-hCG (24).

Assuming that G-CSF has positive effects on pregnancy rate of patients suffering from RM, Wurfel et al. conducted a similar pilot study to evaluate this hypothesis on the rate of pregnancy in patients undergoing ICSI and IVF and their results showed 43% of G-CSF treated patients in comparison with 20% pregnancy rate amongst placebo group (16).

In 2013, Zeyneloglu et al. designed a study to compare the effectiveness of intrauterine injection of G-CSF and subcutaneous injection of this growth factor on pregnancy rate amongst patients undergoing IVF and suffering from RIF. This preliminary report suggested that G-CSF should be considered as a promising and safe agent which increases the rate of pregnancy (27).

Our results also showed that intrauterine injection of G-CSF may decrease the rate of miscarriage but because of non-significant difference between the two study groups, assessment of a larger group of women suffering from RM should be considered. Based on the study’s findings, G-CSF should not be used continuously and only two injections may not be enough.

A retrospective study on 127 RM patients was conducted by Santjohanser et al. with the aim of clarifying the effect of G-CSF treatment among RM subjects undergoing IVF or ICSI. Results showed that G-CSF might be effective on RM but there are several concerns originated from heterogeneous pathology of RM (15).

Although it seems that according to our results G-CSF injection helps the development of fetal heart, but the differences were not statistically significant. These results might change with assessing larger population but there is a possibility that G-CSF injection does not benefit women with history of unexplained recurrent miscarriage.

Colony stimulating factor-1 (CSF-1) also has been used previously for adjuvant therapy against poor treatment response to controlled ovarian hyperstimulation (COH). The results of Takasaki et al.’s study revealed that concurrent use of CSF-1 and regular ovulation stimulator (hMG/hCG) amended the process of follicle development (22). Knowing the fact that immunologic immunologic factors may play a significant role in pathogenesis of unexplained RM, several animal studies have been conducted to test immunotherapy against RM but there are no proven beneficial effects reported ( 28–30).

Because of the novelty of our study, comparing our study’s results with other studies is not possible.

In other studies, the effect of intrauterine G-CSF was evaluated in patients with thin endometrium or RIF. Administration of G-CSF in patients with recurrent pregnancy loss has been studied only in terms of multiple doses of subcutaneous injection. It is suggested that other studies with more patients and repeated doses of intrauterine G-CSF injection be designed for better evaluation.

## Conclusion

Finally, in contrast to possible effect of G-CSF on improvement of implantation rate revealed by some other studies, based on the result of the present study, intrauterine injection of G-CSF could not be suggested for improvement of clinical pregnancy rate and reduction of abortion among patients with unexplained RM. What seem to be of great importance are the changes that must be made in categorization stage of RM patients for more precise detection of women who will benefit from G-CSF treatment. However, further studies with larger sample size, categorization of patient and different doses of G-CSF injection are also needed to clarify the mechanism with which G-CSF affects the pregnancy process.
